# Supplementary material for: Loss of Hippo signaling causes transdifferentiation of neural retina between the optic fissure edges causing coloboma
Source: bioRxiv. 2026 Mar 17:2026.03.13.711620. Preprint. [Version 1] doi: 10.64898/2026.03.13.711620 (PMC13015408; doi:10.64898/2026.03.13.711620)
Supplement: Supplement 2 [file NIHPP2026.03.13.711620v1-supplement-2.pdf]

bioRxiv preprint doi: https://doi.org/10.64898/2026.03.13.711620; this version posted March 17, 2026. The copyright holder for this preprint (which was not certified by peer review) is the author/funder. This article is a US Government work. It is not subject to copyright under 17 USC 105 and is also made available for use under a CC0 license.

Supplementary Figures

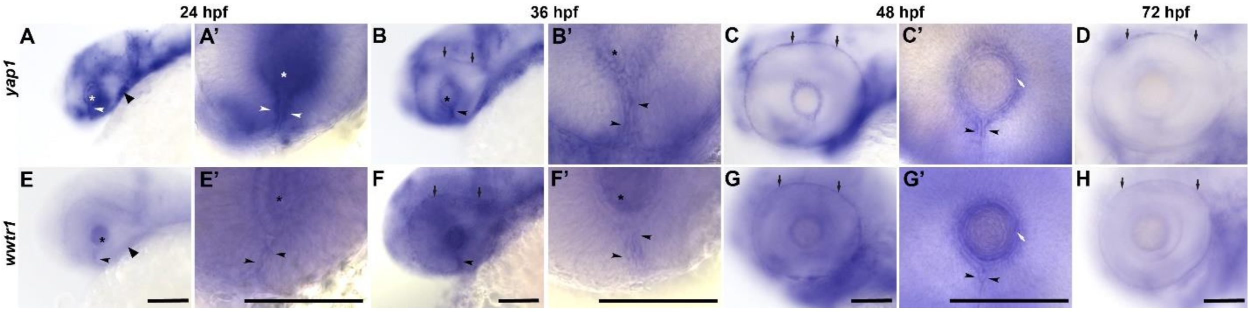

Supplementary Fig. 1: Expression of *yap1* and *wwtr1* in zebrafish eye at various developmental stages: *yap1* and *wwtr1* are expressed in the OF (arrowhead), lens (Asterix) and periocular mesenchyme cells (closed arrowhead) at 24 hpf (A, A', E, E'). By 36 hpf (B, B', F, F') expression is seen in the RPE (arrows) in addition to OF (possibly in pioneer cells, arrowheads) and lens. At 48 hpf, expression at the OF recedes close to lens; becomes prominent in the RPE (C, D), and the expression in lens becomes more restricted to lens epithelium (white arrows in C' and G'). By 72 hpf its expression in the lens epithelium is lost and is only present in the RPE (D, H). A', B', C', E', F', G' are higher magnification images of the optic fissure area of A, B, C, E, F, G respectively. Scale bar is 100 μm. Anterior towards left.

bioRxiv preprint doi: https://doi.org/10.64898/2026.03.13.711620; this version posted March 17, 2026. The copyright holder for this preprint (which was not certified by peer review) is the author/funder. This article is a US Government work. It is not subject to copyright under 17 USC 105 and is also made available for use under a CC0 license.

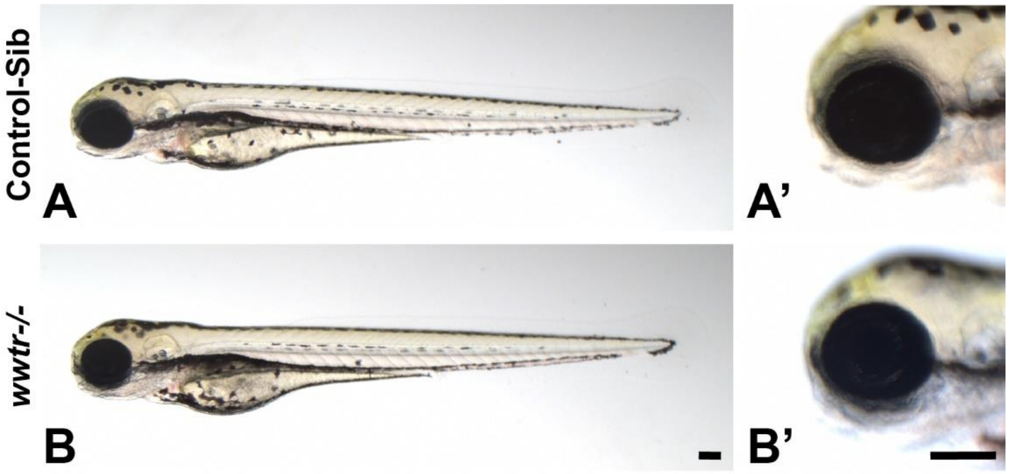

Supplementary Fig. 2: *wwtr1-/-* mutants do not have ocular or pigmentation defects: Phenotypic difference was not observed between *wwtr1-/-* (B, B') compared to control-sib at 72 hpf (A, A'). Scale bar is 100 μm. Phenotype images were taken at 72 hpf.

bioRxiv preprint doi: https://doi.org/10.64898/2026.03.13.711620; this version posted March 17, 2026. The copyright holder for this preprint (which was not certified by peer review) is the author/funder. This article is a US Government work. It is not subject to copyright under 17 USC 105 and is also made available for use under a CC0 license.

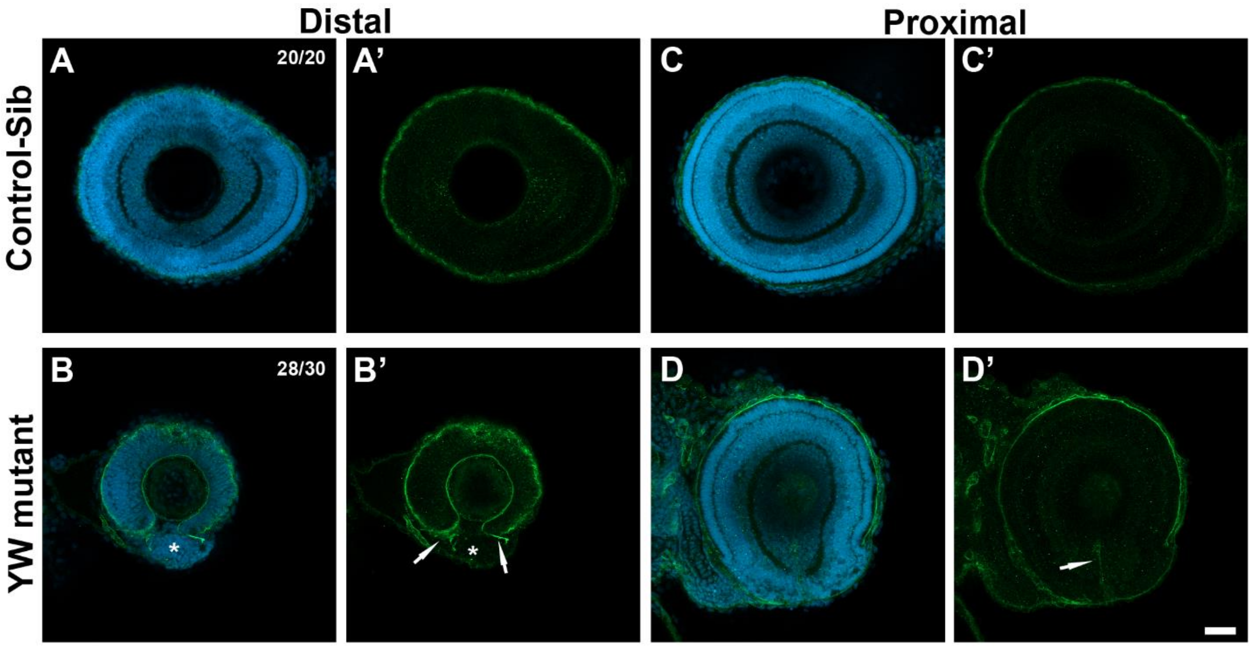

Supplementary Fig. 3: Failure of OF closure in YW mutant embryos is due to a possible tissue hinderance: Basement membrane is breached as indicated by the absence of laminin staining in the distal and proximal sections and OF is fused in the control-sib at 72 hpf (A-C'). However, laminin staining in YW mutants delineate clearly visible optic fissure edges (arrows) with a tissue between the edges (Asterisk) in the distal sections (B- D'). Scale bar is 20 μm.

bioRxiv preprint doi: https://doi.org/10.64898/2026.03.13.711620; this version posted March 17, 2026. The copyright holder for this preprint (which was not certified by peer review) is the author/funder. This article is a US Government work. It is not subject to copyright under 17 USC 105 and is also made available for use under a CC0 license.

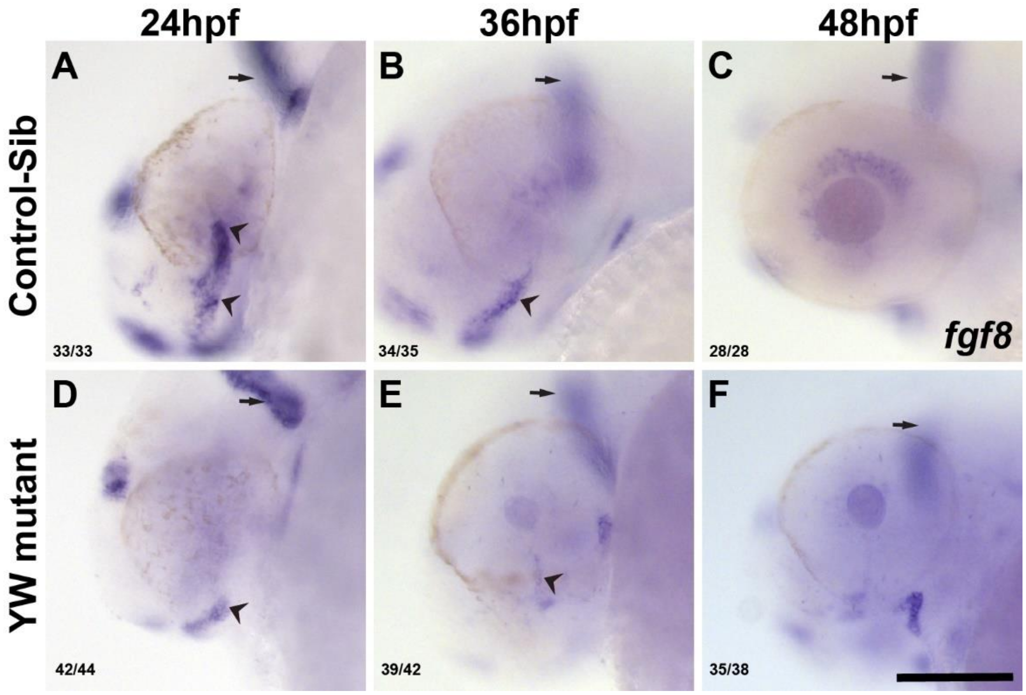

<u>Supplementary Fig. 4: Defects in OS formation in YW mutants</u>: Control-sibs have expression of *fgf8* in the OS at 24 hpf (A) and 36 hpf (B); by 48 hpf its expression in the OS is reduced and can be seen in the retina (C). YW mutant embryos, by contrast, demonstrated reduced expression of *fgf8* at 24 hpf (D), minimal expression levels at 36 hpf (E) and faint expression at the OS and retina at 48 hpf (F). Arrows and arrow heads point to the expression of *fgf8* expression in the brain and OF respectively. Scale bar is 100 μm.

bioRxiv preprint doi: https://doi.org/10.64898/2026.03.13.711620; this version posted March 17, 2026. The copyright holder for this preprint (which was not certified by peer review) is the author/funder. This article is a US Government work. It is not subject to copyright under 17 USC 105 and is also made available for use under a CC0 license.

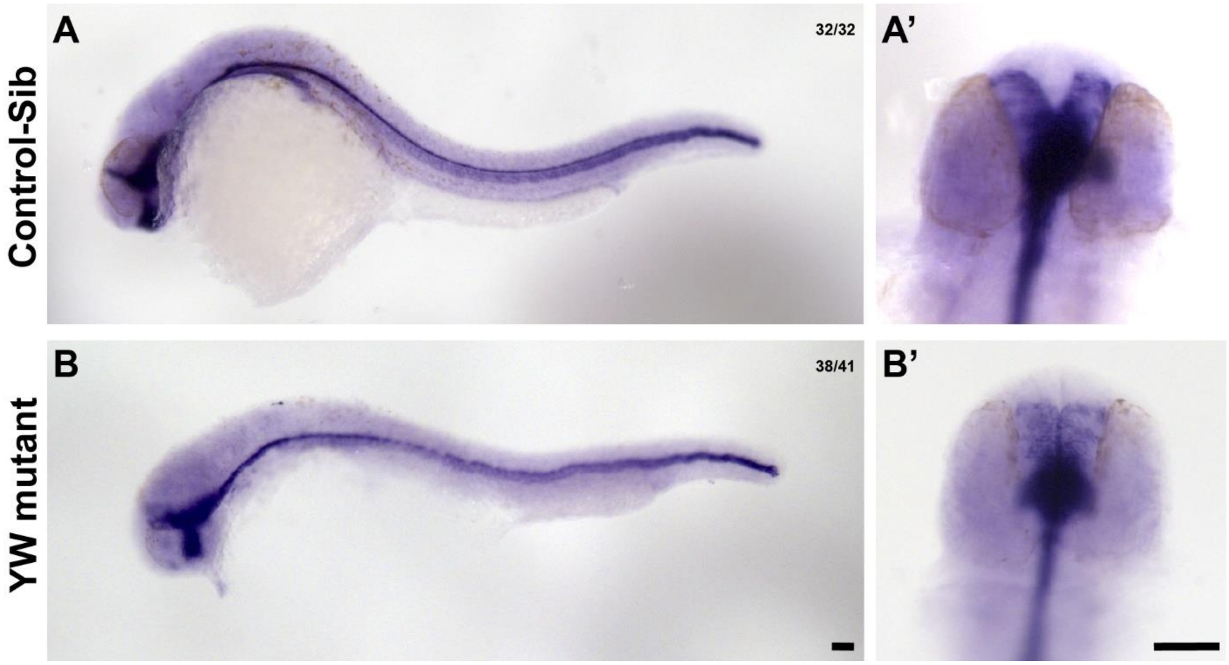

<u>Supplementary Fig. 5: Midline expression of *shha* is not affected in the YW mutants</u>: Midline expression of *shha* in the control-sib is similar to the YW mutants at 24 hpf (A-B'). Scale bar is 100 µm.

bioRxiv preprint doi: https://doi.org/10.64898/2026.03.13.711620; this version posted March 17, 2026. The copyright holder for this preprint (which was not certified by peer review) is the author/funder. This article is a US Government work. It is not subject to copyright under 17 USC 105 and is also made available for use under a CC0 license.

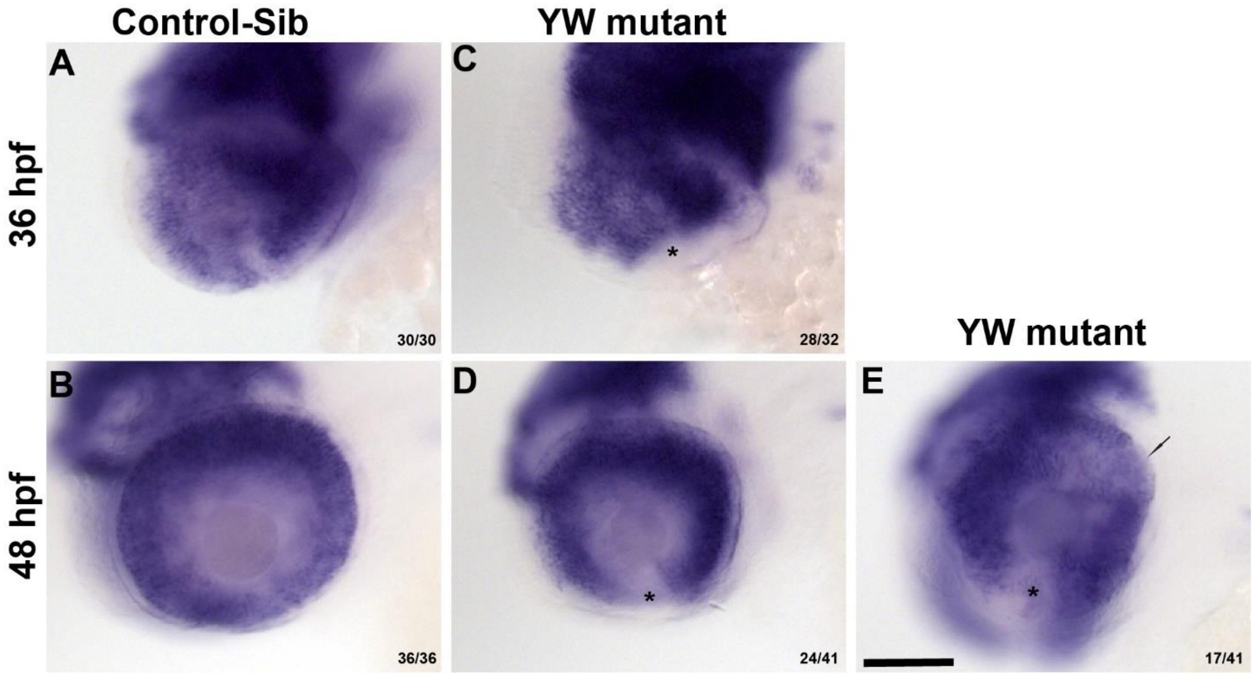

Supplementary Fig. 6: RPE specific Transcription factor *otx2a* is not expressed at the OF in YW mutant embryos: *otx2a* is expressed throughout the OC in 36 hpf and 48hp in control-sib embryos (A, B). YW mutants at 36 hpf exhibit either: 1) broad *otx2a* expression with reduced expression at the OF edges (C), which becomes evident as a gap by 48 hpf (D, Asterix) or 2) patchy expression of *otx2a* in the OC with reduced OF expression (E, arrow). Scale bar is 100 µm.

bioRxiv preprint doi: https://doi.org/10.64898/2026.03.13.711620; this version posted March 17, 2026. The copyright holder for this preprint (which was not certified by peer review) is the author/funder. This article is a US Government work. It is not subject to copyright under 17 USC 105 and is also made available for use under a CC0 license.

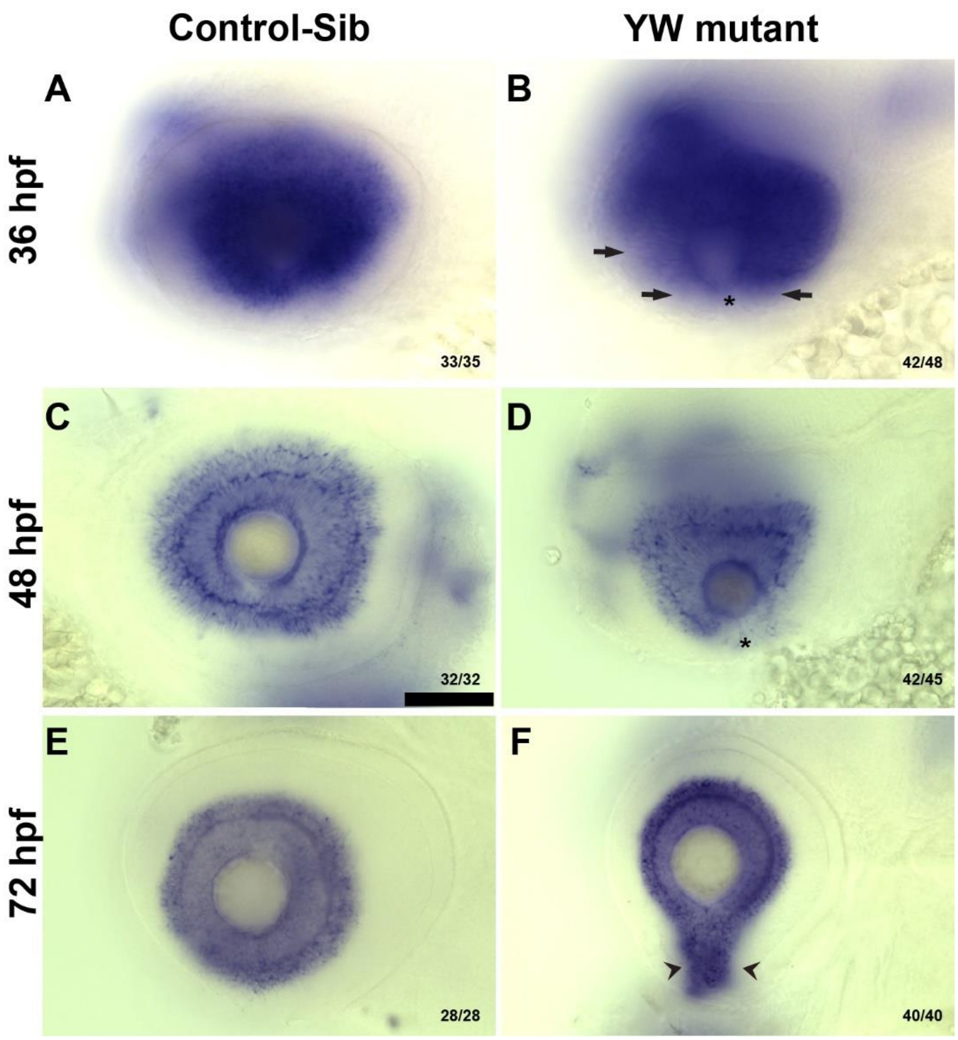

Supplementary Fig. 7: *pax6*, an early marker of the bipotential OC is mis-expressed in the YW mutants: *pax6* is expressed in the ganglion and amacrine cells and their processes at 36 hpf (A), 48 hpf (C) and 72 hpf (E) in the control-sib. YW mutants, have *pax6* expression all over the OC, no expression is observed at the OF and patchy expression on the sides on the OC at 36 hpf (B, Asterix, arrows). Expression of *pax6* is not observed at OF edges and the temporal lobe of the OC have patchy expression at 48 hpf (D, Asterix) and by 72 hpf, the mutants regain expression which extends into the open OF (F, arrowheads). Scale bar is 100 μm.

bioRxiv preprint doi: https://doi.org/10.64898/2026.03.13.711620; this version posted March 17, 2026. The copyright holder for this preprint (which was not certified by peer review) is the author/funder. This article is a US Government work. It is not subject to copyright under 17 USC 105 and is also made available for use under a CC0 license.

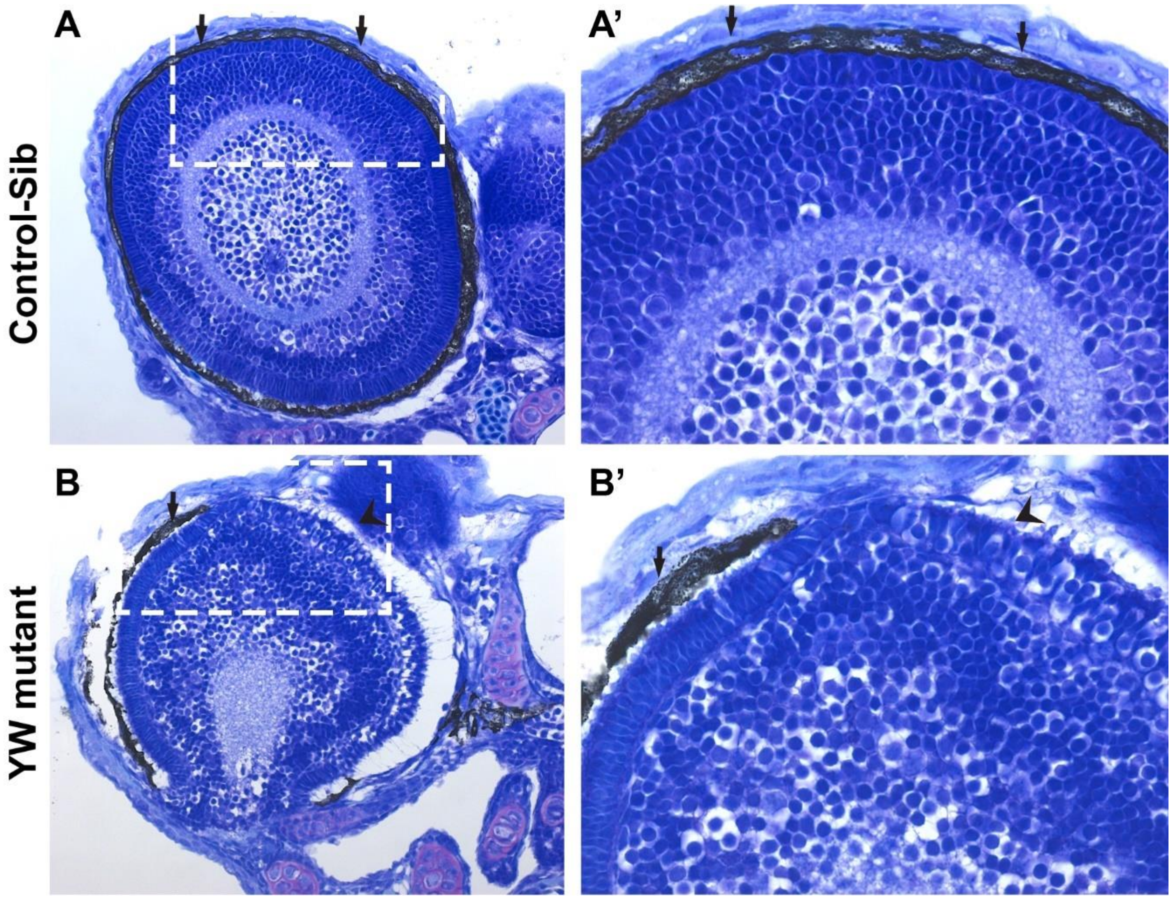

Supplementary Fig. 8: Patchy RPE defects in YW mutant embryos is accompanied by abnormal NR lamination: Histology of the OC at 72 hpf show a well formed RPE and NR in a control-sib (A, A'). However, in YW mutant embryos, retinal lamination is affected and RPE formation is patchy. In the regions of OC having pigment defects, photoreceptor formation is affected (B, B'). A' and B' are high magnification of boxed area in the A and B respectively. Arrows point to areas of retina having RPE and arrowhead to the area without RPE. Scale bar is 50 μm.
